# Red cell distribution width to lymphocyte ratio could serve as a new inflammatory biomarker for predicting hematoma expansion in patients with intracerebral hemorrhage

**DOI:** 10.1186/s12883-024-03669-1

**Published:** 2024-05-15

**Authors:** Milad Babaei Guilan, Seyed Reza Bagheri, Rezvan Roshani, Ehsan Alimohammadi

**Affiliations:** 1 Department of Neurosurgery, Kermanshah University of Medical Sciences, Kermanshah, Iran; 2 Department of neurosurgery, Kermanshah University of Medical Sciences, Imam Reza hospital, Kermanshah, Iran; 3 Clinical Research Development Center, Taleghani and Imam Ali hospital, Kermanshah University of Medical Sciences, Kermanshah, Iran

**Keywords:** Hematoma expansion, Intracerebral hemorrhage, Red cell distribution width to lymphocyte ratio, Hematoma volume

## Abstract

**Background:**

Hematoma expansion is a critical factor associated with increased mortality and adverse outcomes in patients with intracerebral hemorrhage (ICH). Identifying and preventing hematoma expansion early on is crucial for effective therapeutic intervention. This study aimed to investigate the potential association between the Red cell distribution width to lymphocyte ratio (RDWLR) and hematoma expansion in ICH patients.

**Methods:**

We conducted a retrospective analysis of clinical data from 303 ICH patients treated at our department between May 2018 and May 2023. Demographic, clinical, radiological, and laboratory data, including RDWLR upon admission, were assessed. Binary logistic regression analysis was employed to determine independent associations between various variables and hematoma expansion.

**Results:**

The study included 303 ICH patients, comprising 167 (55.1%) males and 136 (44.9%) females, with a mean age of 65.25 ± 7.32 years at admission. Hematoma expansion occurred in 73 (24.1%) cases. Multivariate analysis revealed correlations between hematoma volume at baseline (OR, 2.73; 95% CI: 1.45 -4,78; *P* < 0.001), admission systolic blood pressure (OR, 2.98 ; 95% CI: 1.54–4.98; *P* < 0.001), Glasgow Coma Scale (GCS) (OR, 1.58; 95% CI: 1.25–2.46; *P* = 0.017), and RDWLR (OR, 1.58; 95% CI: 1.13–2.85; *P* = 0.022) and hematoma expansion in these patients.

**Conclusions:**

Our findings suggest that RDWLR could serve as a new inflammatory biomarker for hematoma expansion in ICH patients. This cost-effective and readily available biomarker has the potential for early prediction of hematoma expansion in these patients.

## Background

Intracranial hemorrhage (ICH), the second most prevalent type of stroke, is linked to significant rates of mortality and morbidity. Hematoma expansion occurs in around 30% of ICH cases within the initial 24 h and is correlated with unfavorable neurological outcomes [1–4]. Therefore, early detection and prevention of hematoma expansion are crucial therapeutic goals.

Various factors, such as hematoma size and location, elevated systolic blood pressure, coagulopathy, and systemic inflammatory response syndrome during hospitalization, have been identified as predictors of hematoma expansion [5, 6]. Studies have suggested that absolute and differential leukocyte counts can serve as markers for central nervous system inflammation. The inflammatory response can trigger a cascade of neurochemical events, including changes in cerebral blood flow, blood-brain barrier breakdown, impaired brain tissue metabolism, and cellular damage [2, 7].

The red cell distribution width (RDW) is a parameter included in the complete blood count that reflects the variability in the distribution of red blood cell volumes in circulation. Elevated RDW levels may indicate underlying conditions such as chronic systemic inflammation, inadequate nutrition, and impaired microcirculation [8]. In various vascular diseases, including acute myocardial infarction, symptomatic chronic heart failure, and ischemic stroke, elevated RDW has been identified as a prognostic indicator [9–13]. Furthermore, studies have demonstrated a connection between RDW levels and the development of delayed cerebral ischemia, as well as a poorer prognosis in patients with acute non-traumatic subarachnoid hemorrhage [13, 14]. Lymphocytes play a crucial role in adaptive immune responses.The inflammatory response and immune system work together in the disease progression, leading to conditions such as leukocytosis and lymphocytopenia. T-lymphocytes contribute significantly to the repair of damaged brain tissues through the release of growth factors and the regulation of functions [15]. A reduction in lymphocyte levels following traumatic brain injury (TBI) is viewed as an indicator of brain injury associated with unfavorable clinical outcomes [16]. This study aimed to investigate the potential association between the Red cell distribution width to lymphocyte ratio (RDWLR) and hematoma expansion in ICH patients.

## Methods

We conducted a retrospective investigation of all consecutive patients with spontaneous intracerebral hemorrhage (ICH) admitted to our center between May 2018 and May 2023. We specifically included patients with primary spontaneous ICH who had undergone at least two head CT scans within the first 24 h of admission. Patients under 18 years old at admission, those with secondary causes of ICH (e.g., trauma, aneurysms, tumors, and arteriovenous malformations), a history of anticoagulant medication use, or conditions associated with leukocytosis (such as infection and hematologic malignancies) were excluded from the study. Approval for this study was obtained from the Scientific Research Board of the Kermanshah University of Medical Sciences.

Demographic, clinical, radiological, and laboratory data were gathered from hospital medical records. The location of the hematoma was determined based on the initial brain CT scans and categorized as lobar, deep, cerebellar, or brain stem. Hematoma volume was calculated using the ellipsoid formula (4/3 π a × b × c), where a, b, and c represent the respective radii in 3-dimensional neuroimaging [2].

Hematoma expansion was defined as relative enlargement > 33% or absolute growth > 6 mL [3]. Clinical outcomes at the time of hospital discharge were assessed using the Glasgow Outcome Scale (GOS), which measures global functioning with five outcome categories. We classified the GOS groups into binary categories: favorable (GOS 4,5) and unfavorable (GOS 1,2,3).

Blood sampling was conducted upon admission, and neutrophil and lymphocyte counts were obtained from peripheral hemogram analyses using venous blood samples and an automated blood counter (XN-10, Sysmex Inc., Japan). The red cell distribution width to lymphocyte ratio (RDWLR) was calculated by dividing the red cell distribution width by the lymphocyte count.

## Statistical analysis

The data was presented as mean ± standard deviation. The normality of quantitative variables was evaluated using the Kolmogorov-Smirnov test. The independent t-test, Chi-square test, and Fisher’s exact test were used to compare variables between groups. For data that did not meet the normality assumption, non-parametric tests like the Mann-Whitney U test (for comparing two independent groups), the Kruskal-Wallis test (for comparing more than two independent groups), and the Wilcoxon signed-rank test (for paired data) were utilized for analysis. Binary logistic regression analysis was conducted to explore independent relationships between variables and hematoma expansion. The receiver operating characteristic (ROC) curve with RDWLR values as the test variable in predicting hematoma expansion) was constructed.Data analysis was carried out using SPSS 23 software (SPSS Inc., Chicago, Illinois), with significance set at P values < 0.05.

## Results

We examined a total of 303 patients diagnosed with intracerebral hemorrhage (ICH). Among them, 167 (55.1%) were male and 136 (44.9%) were female. The average age at admission was 65.25 ± 7.32 years. Hematoma expansion was observed in 73 (24.1%) cases. The detailed characteristics of the patient sample can be found in Tables 1 and 2.

**Table 1:** Frequency and frequency percent of the variables

Variable		Number	(%)
Hematoma expansion	Yes	73	24.1
	No	230	75.9
Gender	Male	167	55.1
	Female	136	44.9
Hypertension	Yes	179	59.1
	No	124	40.9
Diabetes	Yes	83	27.4
	No	220	72.6
Smoking	Yes	90	29.7
	No	213	70.3
Hematoma Location	Lobar	131	43.2
	Deep	101	33.3
	Cerebellar	47	15.5
	Brain Stem	24	7.9
GOS	Death	51	16.8
	Vegetative State	27	8.9
	Severe Disability	55	18.2
	Moderate Disability	98	32.3
	Good Recovery	72	23.8
Need For Surgery	Yes	80	26.4
	No	223	73.6
Intera-Ventricular Hemorrhage	Yes	67	22.1
	NO	236	77.9
Hydrocephalus	Yes	42	13.9
	No	261	86.1

**Table 2:** Mean and standard deviation of quantitative variables

variable	Mean (SD)
Age (Year)	65. 25 (7.32)
GCS	8.71 (1.63)
Hospital stay (day)	16.01(6.14)
Hematoma volume at baseline (ml)	17.13 (5.22)
Hematoma volume at 24 h (mL)	19.04 (6.21)
Time to baseline CT scan, h	4.33 (1.21)
Time to 24-h CT scan, h	23.2 (1.67)
Admission systolic blood pressure (mm Hg)	153.42 (8.33)
Admission diastolic blood pressure (mm Hg)	87.59 (5.02)
Baseline white blood cell count cells/mm3	9148 (4603)
Baseline Neutrophil count cells/mm3	7121(2867)
Baseline lymphocyte count cells/mm3	1534 (793)
Platelet count cells/mm3	198,089 (8872)
Admission Prothrombin Time	13.43 (1.22)
Admission Partial Thromboplastin Time	32.7 (3.43)
Admission INR	1.2 (0.32)

Patients experiencing hematoma expansion displayed a poorer prognosis compared to those without hematoma expansion (*p* < 0.05) [Table 3]. Analysis from Table 3 indicated a higher incidence of surgery among patients in the hematoma expansion group compared to those in the non-hematoma expansion group (*p* < 0.05) [Table 3].

**Table 3:** Comparison of two groups (hematoma expansion group vs. non-hematoma expansion group) based on qualitative variables

Variable			Hematoma expansion n (%)		Statistical test
			Yes 73 (24.09%)	No 230(75.91%)	
Gender		Male	40 (54.8)	127 (55.2)	*P* = 0.528
		Female	33 (45.2)	103 (44.8)	
Hypertension		Yes	37(50.7)	142(61.7)	*P* = 0.171
		No	36 (49.3)	88(38.3)	
Diabetes		Yes	21(28.8)	62(27.0)	*P* = 0.435
		No	52 (71.2)	168(73.0)	
Smoking		Yes	18(24.7)	72 (31.3)	*P* = 0.306
		No	55(75.3)	158 (68.7)	
Hematoma Location		Lobar	21(28.8)	80(34.8)	*P* = 0.298
		Deep	37(50.7)	94 (40.9)	
		Cerebellar	9(12.3)	38 (16.5)	
		Brain Stem	6 (8.2)	18 (7.8)	
GOS	Unfavorable outcome	Death	17 (23.3)	34 (14.7)	*P* = 0.014
		Vegetative State	8 (10.9)	19 (8.26)	
		Severe Disability	19 (26.0)	36 (15.65)	
	Favorable outcome	Moderate Disability	17 (23.3)	81(35.2)	
		Good Recovery	12 (16.4)	60 (26.08)	
Need For Surgery		Yes	35 (47.9)	45 (19.6)	*P* = 0.006
		No	38 (52.1)	185 (80.4)	
Intera-Ventricular Hemorrhage		Yes	15 (20.5)	52(22.6)	*P* = 0.424
		No	58 (79.5)	178 (77.4)	
Hydrocephalus		Yes	9 (12.3)	33(14.3)	*P* = 0.846
		No	64(87.7)	197 (85.7)	

Univariate analysis revealed associations between Glasgow Coma Scale (GCS), baseline hematoma volume, admission systolic blood pressure, red cell distribution width (RDW), baseline lymphocyte count, and RDWLR with hematoma expansion in ICH patients (*p* < 0.05) [Tables 3 and 4].

**Table 4:** Comparison of two groups (hematoma expansion group vs. non-hematoma expansion group) based on quantitative variables

variable	Hematoma expansion		Hematoma expansion
	Yes (*n* = 73)	No (*n* = 230)	
Age (Year)	66.31 (4.22)	65.33 (4.13)	*P* = 0.349
GCS	6.78 (1.44)	9.32 (1.76)	*P* = 0.034
Hospital stay (day)	20.01(5.01)	13.54 (4.01)	*P* = 0.029
Hematoma volume at baseline (ml)	24.18 (5.36)	16.12 (5.55)	*P* = 0.011
Admission systolic blood pressure (mm Hg)	170.36 (8.89)	154.11 (7.52)	*P* = 0.023
Admission diastolic blood pressure (mm Hg)	88.96 (5.78)	85.88 (6.13)	*P* = 0.542
Baseline white blood cell count cells/mm3	10,353 (4624)	9673 (4714)	*P* = 0.321
Baseline Neutrophil count cells/mm3	7854 (3221)	7386 (3147)	*P* = 0.278
Baseline lymphocyte count cells/mm3	1273 (562)	1483 (601)	*P* = 0.021
Red cell distribution width, %	15.84 (0.059)	13.24 (0.53)	*P* = 0.018
Red blood cell (million cells/microlitre)	4.56 (0.63)	4.52 (0.62)	*P* = 0.251
Red cell distribution width to lymphocyte ratio	0.012 (0.003)	0.008 (0.001)	*P* = 0.009
Mean corpuscular volume (femtolitres)	86.32 (8.23)	84.73 (7.57)	*P* = 0.423
Mean corpuscular hemoglobin. (picograms/cell)	28.35 (3.22)	27.98 (3.11)	*P* = 0.309
Mean corpuscular hemoglobin concentration(gms/dl)	33.21 (1.84)	33.01 (1.83)	*P* = 0.374
Platelet count cells/mm3	196,573 (8436)	199,842 (8768)	*P* = 0.491
Admission Prothrombin Time	13.12 (1.23)	13.43(1.19)	*P* = 0.554
Admission Partial Thromboplastin Time	34.01 (2.73)	36.75 (3.01)	*P* = 0.762
Admission INR	1.2 (0.41)	1.1(0.37)	*P* = 0.265

Multivariate analysis demonstrated correlations between baseline hematoma volume (OR, 2.73; 95% CI: 1.45–4.78; *P* < 0.001), admission systolic blood pressure (OR, 2.98; 95% CI: 1.54–4.98; *P* < 0.001), Glasgow Coma Scale (GCS) (OR, 1.58; 95% CI: 1.25–2.46; *P* = 0.017), and RDWLR (OR, 1.58; 95% CI: 1.13–2.85; *P* = 0.022) with hematoma expansion in these patients [Table 5].

**Table 5:** Binary logistic regression analysis of hematoma expansion following intracerebral hemorrhage

Variables	Odds ratio	95% CI	*P*-value
Hematoma volume at baseline (ml)	2.73	1.45–4.78	*P* < 0.001
Admission systolic blood pressure (mm Hg)	2.98	1.54–4.98	*P* < 0.001
GCS	1.83	1.25–2.46	*P* = 0.017
Red cell distribution width to lymphocyte ratio	1.58	1.13–2.85	*P* = 0.022
Lymphocyte count cells/mm3	1.26	0.80–1.63	*P* = 0.271
Red cell distribution width, %	1.41	0.92–2.07	*P* = 0.438

**Fig. 1:**
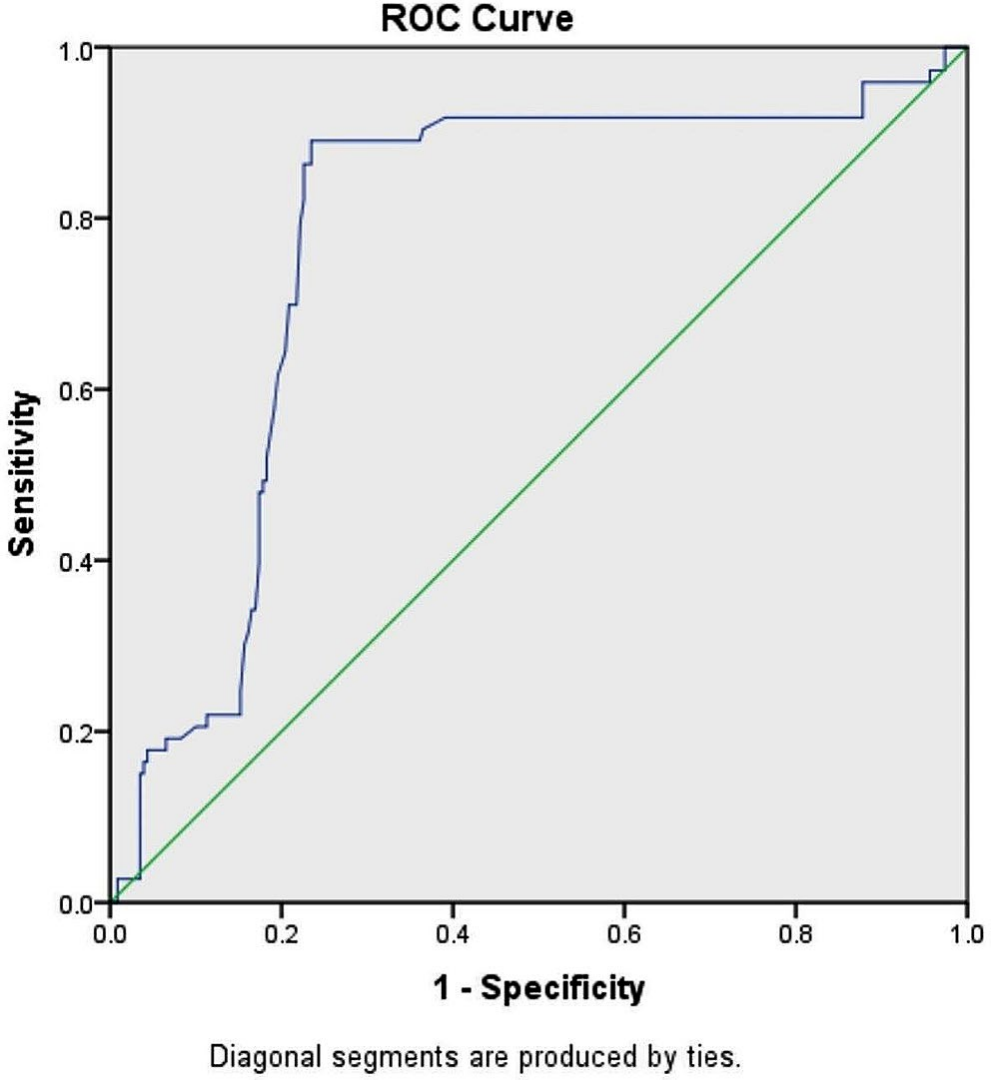
The receiver operating characteristic (ROC) curve with Red cell distribution width to lymphocyte ratio (RDWLR) values as the test variable in predicting hematoma expansion

In Fig. 1, the ROC curve was generated to differentiate between individuals with hematoma expansion and those with no hematoma expansion based on RDWLR values.

## Discussion

The findings of this study suggest a potential association between baseline RDWLR and 24-hour hematoma expansion following intracerebral hemorrhage (ICH). Research indicates that the inflammatory reaction post-ICH can trigger peripheral leukocytosis. The hemorrhage induces microglial activation, leading to the release of cytokines and chemokines that facilitate leukocyte infiltration within a short timeframe [17, 18].

Several studies have explored the relationship between RDW and the clinical outcomes of patients with ICH [11–13].

In a retrospective study by He et al., the relationship between red cell distribution width (RDW) and long-term mortality in patients with intracerebral hemorrhage (ICH) was examined [12]. The study involved 4223 ICH patients. After accounting for potential influencing factors, both the RDW coefficient of variation (RDW-CV) at admission (Quartile 4 [Q4] vs. Quartile 1 [Q1], adjusted hazard ratio [HR] 1.61, 95% confidence interval [CI] 1.34–1.92) and the median RDW-CV within the first month post-admission (Q4 vs. Q1, adjusted HR 1.69, 95% CI 1.40–2.04) were linked to a higher risk of 1-year mortality post-ICH. Similar results were observed for RDW standard deviation (RDW-SD).

Several studies have indicated that astrocytes release extracellular vesicles that modulate the peripheral leukocyte response during brain inflammation. The inflammatory response can initiate a series of neurochemical cascades, resulting in changes in cerebral blood flow, disruption of the blood-brain barrier, impairment of brain tissue metabolism, and cellular damage [2, 7, 19].

The precise mechanisms underlying why RDWLR serves as a predictor of hematoma expansion remain unclear. Various pathophysiological mechanisms have been proposed by researchers, including the link between higher RDW and decreased erythrocyte deformability leading to impaired microcirculation flow, nutritional deficiencies, chronic systemic inflammation, and oxidative stress [8, 20].

Multiple studies have consistently demonstrated a strong correlation between RDW and age as well as disease burden. Patel and colleagues have suggested that the rise in RDW levels could indicate dysfunction in multiple physiological systems associated with the aging process. It has been theorized that elevated erythropoietin levels in aging individuals may act as a compensatory response to subclinical blood loss, reduced red blood cell lifespan, and increased resistance of red cell precursors to erythropoietin [20, 21].

Another potential explanation for the association between increased RDW and aging is the diminished survival of red blood cells due to heightened oxidative stress, a phenomenon observed in conditions characterized by accelerated aging such as Down syndrome [17].

The involvement of lymphocytes in the acute expansion of traumatic intracerebral hemorrhage (tICH) remains uncertain [18, 22]. Leukocytes have the ability to interact with platelets, endothelium, and coagulation factors, suggesting a potential significant role in the pathophysiology of hematoma expansion by influencing the coagulation system [23]. In line with prior research, the current study indicates a decrease in lymphocyte levels in patients experiencing tICH expansion, likely due to a reduction in T lymphocytes [17, 23]. The decline in T lymphocyte numbers is linked to significant deterioration in neurological outcomes and an increased risk of pulmonary infections in traumatic brain injury patients [24]. Given the presence of various subtypes with bidirectional immunomodulatory functions, the potential impact of T lymphocytes in the acute tICH expansion may be complex. Given the proinflammatory conditions during the initial phases of cerebral contusion [25], it is hypothesized that anti-inflammatory T lymphocyte subtypes such as regulatory T (Treg) and Th2 cells could be depleted, leading to a proinflammatory immune response. However, further clinical investigations are necessary to delve into the role of T lymphocytes in the acute tICH expansion and the long-term outcomes following cerebral contusion [25, 26]. The RDWLR, an inflammatory marker, could serve as a straightforward indicator of the interplay between innate and adaptive immunity. In patients with acute cerebral contusion, RDWLR offers a convenient parameter for evaluating an individual’s neuroinflammatory status.

Neuroinflammation, a pivotal aspect of acute cerebral contusion linked to tICH expansion, can impact the progression of the condition and potentially serve as a target for intervention. Neuroinflammation typically initiates following the onset of cerebral contusion [18, 26]. The mechanical injury causes tissue shearing and microvessel fracturing, leading to the formation of an initial hematoma. Danger-associated molecular patterns from components of the hematoma trigger innate immune responses by activating astrocytes and microglia. These cells release various proinflammatory cytokines and chemokines to recruit peripheral monocytes/macrophages and neutrophils. These circulating white blood cells further activate inflammatory pathways, such as the NF-κB signal, which contributes to the induced necrotic death of vascular endothelial cells. This process leads to delayed microvessel fragmentation around the initial hematoma and subsequent expansion of tICH [27, 28].

Moreover, elevated levels of matrix metalloproteinases (MMPs) post-ICH contribute to delayed tICH expansion by promoting the loss of vascular integrity, thereby increasing vascular wall permeability. MMPs also facilitate blood-brain barrier disruption and enhance monocyte and neutrophil infiltration [2, 18]. Consequently, the formation of ICH and leukocyte extravasation into brain tissue amplify reactions, exacerbate cerebral injury in a detrimental cycle, and worsen tICH expansion and edema, ultimately impeding cerebral contusion recovery.

## Limitations

The study has several limitations. It is a single-center retrospective study with a relatively small sample size. The retrospective design introduces potential biases in data selection and analysis, which need to be acknowledged. Furthermore, the assessment of hematoma expansion was limited to the first 24 h, although it is known that hematoma expansion can extend beyond this timeframe. Additionally, data on body temperature and osmotherapy, factors potentially linked to hematoma expansion, were not available. Another limitation of the study is that the potential issue of multicollinearity among the variables included in the multivariate model, such as RDW, lymphocyte count, and RDWLR, was not specifically addressed.

Despite the limitations of this study, it is crucial to acknowledge the potential clinical application of this work. By integrating RDWLR as a predictive biomarker for hematoma expansion, clinicians can proactively identify individuals at a higher risk of adverse outcomes. This early risk assessment could pave the way for tailored treatment strategies, closer monitoring, and timely interventions to mitigate hematoma expansion and its associated complications. Incorporating RDWLR into routine clinical practice has the potential to elevate patient care by facilitating more accurate prognostication and personalized management in ICH cases, ultimately enhancing patient outcomes and potentially lowering mortality rates.

## Conclusions

The results of our study indicate that RDWLR could potentially function as a prognostic indicator for hematoma expansion in patients with ICH. This cost-effective and easily accessible biomarker shows promise for early detection of hematoma expansion in this patient population.

## Data Availability

The datasets generated and/or analysed during the current study are not publicly available due them containing information that could compromise research participant privacy/consent but are available from the corresponding author on reasonable request.
